# Molecular characterisation of *ERG*, *ETV1* and *PTEN* gene loci identifies patients at low and high risk of death from prostate cancer

**DOI:** 10.1038/sj.bjc.6605554

**Published:** 2010-01-26

**Authors:** A H M Reid, G Attard, L Ambroisine, G Fisher, G Kovacs, D Brewer, J Clark, P Flohr, S Edwards, D M Berney, C S Foster, A Fletcher, W L Gerald, H Møller, V E Reuter, P T Scardino, J Cuzick, J S de Bono, C S Cooper

**Affiliations:** 1 The Institute of Cancer Research, Male Urological Cancer Research Centre, Surrey, UK; 2 The Royal Marsden NHS Foundation Trust, Surrey, UK; 3 Wolfson Institute of Preventive Medicine, Barts and The London School of Medicine and Dentistry, Queen Mary University of London, London, UK; 4 Laboratory of Molecular Oncology, Medical Faculty, Ruprecht-Karls-Universitat, Heidelberg, Germany; 5 The Orchid Tissue Bank, Department of Molecular Oncology and Imaging, Institute of Cancer, Barts and the London School of Medicine and Dentistry, London, UK; 6 Department of Pathology, Royal Liverpool University Hospital, Liverpool, UK; 7 Department of Pathology, Memorial Sloan-Kettering Cancer Center, New York, NY, USA; 8 Thames Cancer Registry, King's College London, London, UK

**Keywords:** *ERG/ETV1* gene rearrangements, fluorescence *in situ* hybridisation, *PTEN* gene loss, prostate cancer, survival

## Abstract

**Background:**

The discovery of *ERG/ETV1* gene rearrangements and *PTEN* gene loss warrants investigation in a mechanism-based prognostic classification of prostate cancer (PCa). The study objective was to evaluate the potential clinical significance and natural history of different disease categories by combining *ERG/ETV1* gene rearrangements and *PTEN* gene loss status.

**Methods:**

We utilised fluorescence *in situ* hybridisation (FISH) assays to detect *PTEN* gene loss and *ERG/ETV1* gene rearrangements in 308 conservatively managed PCa patients with survival outcome data.

**Results:**

*ERG/ETV1* gene rearrangements alone and *PTEN* gene loss alone both failed to show a link to survival in multivariate analyses. However, there was a strong interaction between *ERG/ETV1* gene rearrangements and *PTEN* gene loss (*P*<0.001). The largest subgroup of patients (54%), lacking both *PTEN* gene loss and *ERG/ETV1* gene rearrangements comprised a ‘good prognosis’ population exhibiting favourable cancer-specific survival (85.5% alive at 11 years). The presence of *PTEN* gene loss in the absence of *ERG/ETV1* gene rearrangements identified a patient population (6%) with poorer cancer-specific survival that was highly significant (HR=4.87, *P*<0.001 in multivariate analysis, 13.7% survival at 11 years) when compared with the ‘good prognosis’ group. *ERG/ETV1* gene rearrangements and *PTEN* gene loss status should now prospectively be incorporated into a predictive model to establish whether predictive performance is improved.

**Conclusions:**

Our data suggest that FISH studies of *PTEN* gene loss and *ERG/ETV1* gene rearrangements could be pursued for patient stratification, selection and hypothesis-generating subgroup analyses in future PCa clinical trials and potentially in patient management.

Prostate cancer (PCa) is the most commonly diagnosed male cancer and the second commonest cause of male cancer related mortality in the Western world (Ferlay *et al*, 2007). The clinical behaviour and molecular pathology of PCa is highly variable. There is an urgent need to dissect this inter-patient heterogeneity with robust molecular biomarkers to accelerate the successful conduct of clinical trials for this disease, optimise patient treatment and minimise late drug development attrition (Betensky *et al*, 2002; Attard *et al*, 2008a). Critically, identifying patient subgroups that require less treatment from those that should be targeted with more aggressive therapy is a key goal.

*PTEN* loss and *ETS* gene rearrangements are proposed to be critically important and common molecular events in prostate carcinogenesis (Trotman *et al*, 2003; Tomlins *et al*, 2005, 2008a; Carver *et al*, 2009; King *et al*, 2009). In particular, recent publications have addressed the relationship between the two events in mouse models demonstrating cooperation (Carver *et al*, 2009; King *et al*, 2009). Deletion of all or part of the tumour suppressor gene *PTEN* is a frequent event. Other classes of alterations including mutations and post-translational modifications occur less frequently (Whang *et al*, 1998; Verhagen *et al*, 2006; Yoshimoto *et al*, 2007, 2008). Previous studies have examined the prognostic significance of *PTEN* deletions by fluorescence *in situ* hybridisation (FISH) with small patient cohorts and biochemical recurrence as the outcome (Yoshimoto *et al*, 2007, 2008). Similarly the clinical implications of the ETS gene rearrangements (Tomlins *et al*, 2005, 2007) are not yet fully understood (Clark and Cooper, 2009), with their presence reported as associated with both poor (Demichelis *et al*, 2007; Nam *et al*, 2007; Attard *et al*, 2008b) and good prognosis (Petrovics *et al*, 2005; Gopalan *et al*, 2009; Hermans *et al*, 2009). We reported that deletion of the intermediate region between *TMPRSS2* and *ERG* combined with duplication of the *TMPRSS2–ERG* fusion sequences is predictive of poor cancer-specific survival (Attard *et al*, 2008b), an observation supported by other studies (FitzGerald *et al*, 2008; Yoshimoto *et al*, 2008; Gopalan *et al*, 2009). The effect of *PTEN* loss on PCa survival and its relationship to the ETS gene rearrangements is largely unknown.

To evaluate the natural history of the different disease variants identified by *PTEN*, *ERG* and *ETV1* FISH we evaluated a retrospective cohort of conservatively managed men (Cuzick *et al*, 2006). Immunohistochemistry (IHC) was not carried out because of concerns regarding potential interobserver interpretation variability (Kirkegaard *et al*, 2006); lack of uniformity regarding tissue processing as samples were taken from different hospitals (Cuzick *et al*, 2006); and previous studies reporting conflicting results with IHC (Bedolla *et al*, 2007; McCall *et al*, 2008; Sircar *et al*, 2009). Rearrangements involving *ETV4* and *ETV5* have also been reported, but these are rare and therefore unlikely to be used widely in a clinically applicable test (Kumar-Sinha *et al*, 2008).

## Patients and methods

### Patient cohort

Tissue microarrays (TMAs) were constructed from unselected transurethral resection of prostate (TURP) specimens from patients who received no initial treatment in a cohort of conservatively managed men with PCa (Cuzick *et al*, 2006). Ethical approval for the collection of the cohort was obtained from the Ethics Review Committees at every collaborating hospital.

### TMAs and FISH studies

**Figure 1:**
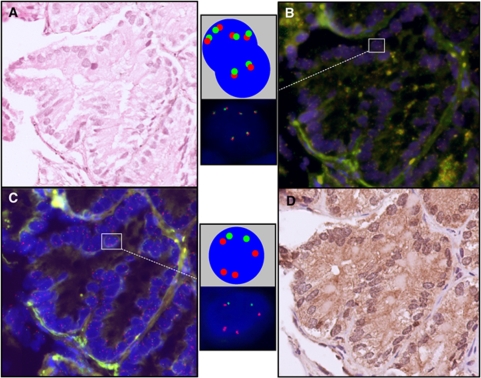
Haematoxylin and eosin (H & E), fluorescence *in-situ* hybridisation (FISH) and P63/alpha-methylacyl-CoA racemase (AMACR) on adjacent slides (**A**) a prostate cancer gland with H & E staining; (**B**) *ETV1* FISH in the same gland on an adjacent slide. Cartoon and magnified images of two nuclei are also shown. The upper nucleus has four paired (ploidy) *ETV1* probes and the lower nucleus has two paired *ETV1* probes indicating wild-type *ETV1*; FISH for *ERG* also showed paired probes indicating wild-type *ERG* (image not shown); (**C**) *PTEN* FISH in the same gland on an adjacent slide. Cartoon and magnified images of one nucleus is shown. The nucleus has four (ploidy) chromosome, 10 centromeric probes (in red) and two *PTEN* probes (in green) indicating heterozygous loss of *PTEN*; and (**D**) the same prostate cancer gland on an adjacent slide, which has absent P63 staining and AMACR positivity.

**Figure 2:**
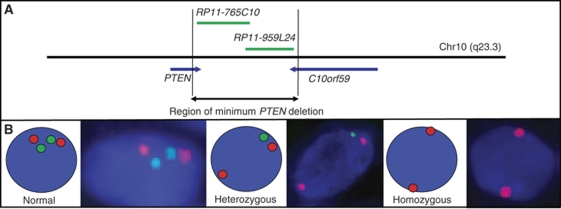
Fluorescence *in-situ* hybridisation (FISH) detection of *PTEN* loss. (**A**) Position of two ‘*PTEN’* BAC probes shown in green. The *PTEN* gene and flanking gene *C10orf59* are shown in dark blue. Arrows indicate the direction of transcription. (**B**) *PTEN* loss patterns. Green signals are probes that detect *PTEN* and red signals are probes that detect the chromosome 10 centromere. Nuclei with normal *PTEN* complement are visualised in interphase as two green and two red signals (left). Heterozygous *PTEN* loss results in the loss of one green signal (centre) and homozygous *PTEN* loss, the loss of two green signals (right).

Tissue microarrays were constructed as previously described (Attard *et al*, 2008b). Areas of ‘cancer’ and ‘normal’ were identified on the basis of histopathological examination of haematoxylin and eosin and p63/AMACR-stained sections that flanked the TMA slice used for FISH studies (Figure 1). To assess the frequency of *PTEN* gene loss in TMA cancer cores, FISH procedures were carried out as described previously for rearrangement at the *ERG* and *ETV1* loci (Attard *et al*, 2008b, 2008c). The same (or an immediately adjacent) slice was stripped and rehybridised for *PTEN*. We selected *PTEN* BACs that map to the minimum region of *PTEN* deletion in PCa as previously described in xenografts and cell lines (Hermans *et al*, 2004). We used two, overlapping, DIG-labelled BAC probes to the 5′ end of the *PTEN* locus, RP11-765C10 and RP11-959L24, and a commercially available CY-3 labelled DNA chromosome 10 centromere probe (chromosome 10 (p11.1∼q11.1 Abbott Molecular, Des Plaines, IL, USA) (Figure 2A). Representative images of nuclei with normal, heterozygous and homozygous *PTEN* patterns are shown in Figures 1 and 2B.

### Establishing *PTEN* cut-offs

Evaluation of the FISH results in each core containing cancer was independently carried out by two operators (A-R and G-A) who were unaware of clinical data. Signals were scored in at least 200 non-overlapping nuclei. We assumed that part of some nuclei can be lost during slicing and therefore not all nuclei in a section would have their full probe complement. Using accepted methodology described previously we estimated the degree of technical artefact by studying nuclei in TMA cores with non-malignant prostatic epithelium (Ventura *et al*, 2006). As cancer nuclei may differ in size from non-malignant nuclei, we also counted patterns of *PTEN* loss in cancer nuclei for which an overall ‘normal *PTEN* complement’ FISH score had been given. These data are provided in Supplementary Figure 1 and Supplementary Table 1.

### Statistical analysis

Associations between *PTEN* status and categorical data were examined using the *χ*^2^ test for trend. Associations between *PTEN* status and numerical variables were assessed using analysis of variance. The primary endpoints for this study were time to death from PCa and time to death from any cause. Univariate and multivariate analyses were carried out by proportional hazard (Cox) regression analysis (Cox and Oakes, 1984). The following variables, determined as described previously (Cuzick *et al*, 2006), were included in the multivariate analyses: centrally reviewed Gleason scores determined by modern grading criteria, baseline PSA (PSA within 6 months of diagnosis) and age at diagnosis. All *P*-values were two-sided and all statistical analyses were carried out using Stata 10.0 (StataCorp, LP, College Station, TX, USA).

## Results

### General

*PTEN* gene status was established from 678 cancer cores from 322 patients. Tissue cores can hybridise with variable efficiency for different FISH probes. Moreover, cores can sometimes be lost in the rehybridisation process. Therefore, *ERG* and *ETV1* gene status was available from 308 of the 322 patients with a PTEN score (662 TMA cancer cores). Demographics and characteristics for tumours stratified for *PTEN* status alone are shown in Table 1 and according to *PTEN* and *ERG/ETV1* gene status shown in Table 2. The median follow-up was 100 months (3–197 months). A competing risk analysis demonstrated that after 11 years of follow-up, 59% of men had died: 25% from PCa and 34% from other causes; only 22% were alive without progression. Results are described for both cause-specific prostate survival and overall survival.

### *PTEN* gene loss alone is not a significant predictor of clinical outcome in multivariate analysis

**Figure 3:**
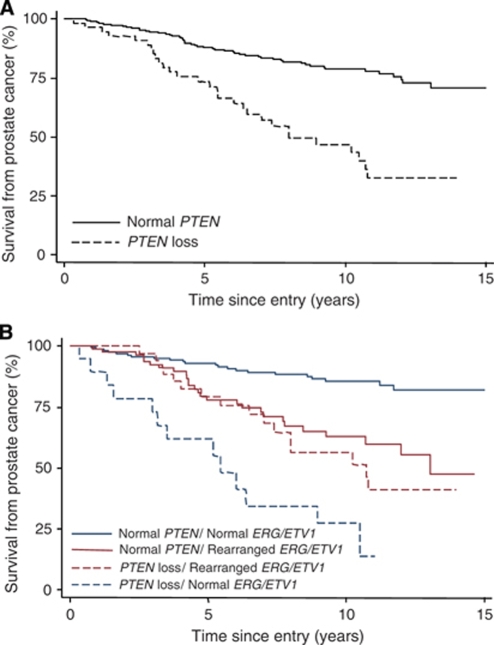
(**A**) Prostate cancer survival according to *PTEN* gene status and (**B**) according to *PTEN* and *ERG/ETV1* gene status.

There was no statistically significant difference in outcome between the tumours with heterozygous and those with homozygous *PTEN* loss (data not shown). Therefore, the analyses presented consider *PTEN* loss tumours as one group. Of the 322 patients with a *PTEN* score, 266 (83%) had a normal *PTEN* score and 56 (17%) had *PTEN* loss. There were significant associations between *PTEN* loss and Gleason score (*P*<0.001), clinical stage (*P*<0.001), baseline PSA (*P*<0.001) and cancer in biopsy (proportion of TURP chips with disease or linear proportion of needle biopsy containing disease) (*P*<0.001), but no association with age (Table 1). Univariate Cox analysis demonstrated that compared to cancers with *PTEN*, tumours with *PTEN* loss had significantly worse cause-specific and overall survival (HR=3.33, 95% CI=2.11–5.26, *P*<0.001, Figure 3A, and HR=1.72, 95% CI=1.24–2.38, *P*=0.001). However, in a multivariate model, *PTEN* loss did not retain significance as a prognostic factor for PCa survival (HR=1.19, 95% CI=0.73–1.96, *P*=0.49) or overall survival (HR=1.00, 95% CI=0.70–1.44, *P*=0.99). Furthermore, even when Gleason score only was added to the model, *PTEN* did not remain a significant independent prognostic factor for PCa survival (HR=1.61, 95% CI=0.99–2.62, *P*=0.05) or overall survival (HR=1.16, 95% CI=0.82–1.65, *P*=0.41).

### *PTEN* gene loss and *ERG/ETV1* gene rearrangements

Univariate Cox analysis demonstrated that *ERG/ETV1* gene rearrangement status alone was a significant prognostic factor for cause-specific and overall survival (HR=2.17, 95% CI=1.39–3.40, *P*=0.001 and HR=1.58, 95% CI=1.20–2.08, *P*=0.001). However, on multivariate Cox analysis *ERG*/*ETV1* gene rearrangement status alone was not a significant independent prognostic factor for either cause-specific or overall survival (HR=0.93, 95% CI=0.58–1.50, *P*=0.78 and HR=1.04, 95% CI=0.77–1.41, *P*=0.80). We next considered *PTEN* and *ERG/ETV1* gene status together (Table 2). The 308 patients were stratified into those that had an *ERG* or *ETV1* gene rearrangement in their cancers (122 patients, 40%) and those that did not (186 patients, 60%). The patients were then further stratified as to whether they had *PTEN* loss or not. There was a significant association between *PTEN* score and *ERG/ETV1* status (*P*<0.001) with 66% of *PTEN* loss tumours also having an *ERG/ETV1* gene rearrangement compared with 34% of normal *PTEN* tumours (Table 3). When the patients were stratified according to their *ERG/ETV1* gene status there was a significant interaction with *PTEN* status (test for heterogeneity (Q (1df)=20.7, *P*<0.001 and Q (1df)=20.9, *P*<0.001 in multivariate analysis for cause-specific survival and overall survival, respectively). Cox analyses were therefore conducted using the group with normal *ERG/ETV1* and normal *PTEN* as the reference.

### *PTEN* gene loss with no *ERG/ETV1* rearrangement identifies a poor prognosis group

Univariate analysis demonstrated that patients with normal *ERG/ETV1* and *PTEN* loss had a significantly worse cause-specific and overall survival (HR=9.37, 95% CI=4.68–18.76, *P*<0.001, Figure 3B and HR=3.14, 95% CI=1.89–5.23, *P*<0.001 respectively). In multivariate analysis cases with normal *ERG/ETV1* and *PTEN* loss had a significantly higher risk of dying from PCa (HR=4.87, 95% CI=2.28–10.41, *P*<0.001). The cancer-specific survival at 11 years was 13.7%. Similar results were found for overall survival (HR=2.40, 95% CI=1.40–4.11, *P*=0.001. Gleason grades of these tumours (Table 2) demonstrate that in 21% of the normal *ERG/ETV1* and *PTEN* loss tumours, Gleason grade was <7 supporting a reclassification of this low Gleason grade patient subgroup.

### *ERG/ETV1* gene rearranged tumours with and without *PTEN* loss form two intermediate prognostic groups

In univariate analysis men with rearranged *ERG/ETV*1 and normal *PTEN* exhibited significantly worse cause-specific and overall survival (HR=2.99, 95% CI=1.70–5.25, *P*<0.001, Figure 3B and HR=1.80, 95% CI=1.30–2.47, *P*<0.001 respectively) compared with men with normal *ERG/ETV1* and *PTEN* status. Similar results were observed for cases with rearranged *ERG/ETV1* and *PTEN* loss (HR=3.92, 95% CI=2.06–7.48, *P*<0.001, Figure 3B and HR=1.77, 95% CI=1.16–2.70, *P*=0.008, respectively). In multivariate analysis, men with rearranged *ERG/ETV1* and normal *PTEN* had a marginally higher risk of dying from PCa (HR=1.82, 95% CI=1.01–3.26, *P*=0.04), but no effect was observed in cases with rearranged *ERG/ETV1* and *PTEN* loss (HR=0.98, 95% CI=0.49–1.97, *P*=0.96). Similar results were found for overall survival (HR=1.37, 95% CI=0.98–1.92, *P*=0.06 and HR=0.84, 95% CI=0.52–1.35, *P*=0.48, respectively). The cancer-specific survival at 11 years for the rearranged *ERG*/*ETV1* and normal *PTEN* group was 59.8% and for the rearranged *ERG/ETV1* and *PTEN* loss group was 41.0% (Figure 3B).

### No *ERG/ETV1* gene rearrangement and no *PTEN* gene loss identifies a good prognosis group

The largest group of patients (*n*=167, 54%) comprised those who had neither an *ERG/ETV1* gene rearrangement nor *PTEN* loss. This group (mean age 69±5 years) had a greater cause specific survival (85.5% at 11 years) and overall survival when compared with the three other groups (Figure 3B). In this good prognosis group, 60 out of 167 (36%) had a Gleason grade ⩾7. Prostate cancer-specific deaths in this cohort were not confined to the higher Gleason grades (Table 2). Of these 20 men who died from PCa at 11 years, 5 (25%) had a Gleason score of <7; 7 (35%) had a Gleason score of 7; and the remaining 8 (40%) had a Gleason score of >7.

## Discussion

We present the first large series in which *PTEN* and *ERG/ETV1* gene status have been analysed together. We have identified patient subgroups with high and low risk of death from PCa based on *PTEN* and *ERG/ETV1* status. There was no difference in outcome between tumours with heterozygous and those with homozygous *PTEN* loss and the *PTEN* loss tumours were therefore considered as one group. This may be because in heterozygous tumours by FISH, the other allele is lost by an alternative mechanism (Whang *et al*, 1998; Verhagen *et al*, 2006). The ‘good prognosis’ group (54%) lacked an *ERG/ETV1* gene rearrangement and *PTEN* gene loss (85.5% PCa survival at 11 years). The PCa-specific deaths in this group did not only occur in the patients with the higher Gleason grades, but across the Gleason grades. These results highlight some inadequacy of Gleason grading in determining which patients require more intensive therapy for their PCa.

We also identified a patient group with a significant ‘poor prognosis’. Patients lacking an *ERG/ETV1* gene rearrangement but with *PTEN* gene loss had the worst cause-specific survival of 13.7% at 11 years. A proportion of patients in this group had a Gleason score of ⩽7. These data are also potentially of clinical importance as they identify a patient group who could be targeted to receive more intensive neoadjuvant and adjuvant therapy when other clinicopathological parameters recommended a more conservative approach. This requires testing in prospectively designed studies. Agents that specifically target the PI3K–AKT–mTOR pathway are undergoing investigation in clinical trials (Yap *et al*, 2008) and future studies should specifically evaluate these agents in this subgroup.

One previous study (82 patients) reported that the absence of *PTEN* loss and no *ERG* gene rearrangement is associated with a longer time to biochemical recurrence and *PTEN* loss plus an *ERG* gene rearrangement was associated with the shortest time to biochemical recurrence (Yoshimoto *et al*, 2008). Our results are similar in that those tumours that had neither an *ERG* gene rearrangement nor *PTEN* loss were in a good prognostic group. However, we did not show that the combination of the two alterations was associated with poor outcome, and rather that it is those tumours with *PTEN* loss but no *ERG/ETV1* gene rearrangement that do particularly badly. These differences may be a consequence of different study sizes or endpoints used.

*ERG/ETV1* gene rearrangement positive and negative tumours have been reported to have distinct chromosomal aberrations, expression signatures and morphological features (Kumar-Sinha *et al*, 2008), thus suggesting that they represent different PCa classes. Our results support this hypothesis because *PTEN* loss was only a significant independent prognostic factor for overall survival, when analysed in the *ERG/ETV1* gene non-rearranged tumours. These results also raise the question of whether there are other molecular abnormalities that are mutually exclusive of *ERG/ETV1* gene rearrangements that may contribute to the worse outcome of patients with non-*ERG/ETV1* gene rearranged/*PTEN* loss tumours. In this respect, the recent observation that a proportion of ETS-gene rearrangement negative cancers overexpress SPINK1 protein is of particular interest (Tomlins *et al*, 2008b). SPINK1 expression was linked to poorer outcome (Tomlins *et al*, 2008b) and SPINK1 has been shown, when overexpressed in colorectal and breast cancer cells, to function as an autocrine growth factor that can stimulate the PI3K pathway (Gouyer *et al*, 2008).

## Conclusions

In conclusion, these data suggest that molecular characterisation of *PTEN*, *ERG* and *ETV1* gene status might be used in future to determine the risk of PCa death. This has implications both for potentially deciding which patients should be conservatively or aggressively treated and also for stratification of patients in clinical trials. At present clinical trial patients are stratified by clinicopathological features alone. Our results suggest that an imbalance in numbers of patients with different *PTEN* and *ERG/ETV1* gene status in different study arms could falsely influence trial outcome and needs to be accounted for.

## Figures and Tables

**Table 1:** Relationship of *PTEN* status with demographics and tumour characteristics

	***PTEN* status**		
	**Normal *PTEN* (*n*=266)**	***PTEN* loss (*n*=56)**	***P*-value^a^**
**Variable**			
Mean age±s.d. (years)	69±5	70±4	0.18
			
*Classes of age (years)*			
⩽65	51	8 (14)	
>65–70	72	13 (15)	
>70–73	72	14 (16)	0.12
>73–76	71	21 (23)	
			
*Gleason score*			
<7	146	5 (3)	
=7	65	18 (22)	<0.001
>7	55	33 (38)	
			
*Baseline PSA (ng ml*^−*1*^)			
⩽4	104	9 (8)	
>4–10	55	7 (11)	
>10–25	51	14 (21)	<0.001
>25–50	31	16 (34)	
>50–100	25	10 (29)	
			
*Clinical stage* b			
T1	81	6 (7)	
T2	50	14 (22)	<0.001
T3	19	19 (50)	
			
*Cancer in biopsy* c			
⩽6	74	2 (3)	
>6–20	75	5 (6)	
>20–40	38	10 (21)	<0.001
>40–75	31	13 (30)	
>75–100	45	26 (37)	

a Test for trend in PTEN loss group (except for mean age).

b Restricted to patients for whom clinical stage is available.

c Restricted to patients for whom extent of disease is available.

**Table 2:** Relationship of *PTEN* and *ERG/ETV1* status with demographics and tumour characteristics

	***PTEN*-*ERG/ETV1* status**			
	**Normal *PTEN*/ normal *ERG/ETV1* (*n*=167)**	**Normal *PTEN*/rearranged *ERG/ETV1* (*n*=85)**	***PTEN* loss/normal *ERG/ETV1* (*n*=19)**	***PTEN* loss/rearranged *ERG/ETV1* (*n*=37)**
**Variable**				
Mean age±s.d. (years)	69±5	69±6	71±4	70±5
				
*Classes of age (years)*				
⩽65	30	18	2	6
>65–70	45	21	5	8
>70–73	48	23	4	10
>73–76	44	23	8	13
				
*Gleason score*				
<7	107	29	4	1
=7	37	27	4	14
>7	23	29	11	22
				
*Baseline PSA (ng ml*^−*1*^)				
⩽4	77	21	7	2
>4–10	35	17	1	6
>10–25	31	17	7	7
>25–50	11	19	3	13
>50–100	13	11	1	9
				
*Clinical stage* a				
T1	58	18	2	4
T2	27	19	5	9
T3	9	8	4	15
				
*Cancer in biopsy* (%)^b^				
⩽6	58	8	2	0
>6–20	51	22	3	2
>20–40	24	13	4	6
>40–75	16	15	2	11
>75–100	16	26	8	18

a Restricted to patients for whom clinical stage is available.

b Restricted to patients for whom extent of disease is available.

**Table 3:** Summary results of multivariate Cox analyses for prostate cancer survival

		**Normal *PTEN***	***PTEN* loss**	**All**
		*n*=167	*n*=19	
	**Normal *ERG/ETV1***	HR=ref	HR=4.87 95% CI=2.28–10.41* P*<0.001	HR=ref
		GS<7: 64%	GS<7: 21%	
		GS=7: 22%	GS=7: 21%	
				
*ERG/ ETV-1* status		*n*=85	*n*=37	
	**Rearranged *ERG/ETV1***	HR=1.82 95% CI=1.01–3.26* P*=0.04	HR=0.98 95% CI=0.49–1.97* P*=0.96	HR=0.93 95% CI=0.58–1.50* P*=0.78
		GS<7: 34%	GS<7: 3%	
		GS=7: 32%	GS=7: 38%	
				
	**All (308 with *ERG/ETV1* status)**	HR=ref	HR=1.12 95% CI=0.68–1.86* P*=0.65	
	**All (322)**	HR=ref	HR=1.19 95% CI=0.73–1.96* P*=0.49	

Abbreviations: CI=confidence interval; GS=Gleason score; HR=hazard ratio.

## References

[bib1] Attard G, Ang JE, Olmos D, de Bono JS (2008a) Dissecting prostate carcinogenesis through ETS gene rearrangement studies: implications for anticancer drug development. J Clin Pathol 61: 891–896 18495790 10.1136/jcp.2008.056341

[bib2] Attard G, Clark J, Ambroisine L, Fisher G, Kovacs G, Flohr P, Berney D, Foster CS, Fletcher A, Gerald WL, Moller H, Reuter V, De Bono JS, Scardino P, Cuzick J, Cooper CS (2008b) Duplication of the fusion of TMPRSS2 to ERG sequences identifies fatal human prostate cancer. Oncogene 27: 253–263 17637754 10.1038/sj.onc.1210640 PMC2646890

[bib3] Attard G, Clark J, Ambroisine L, Mills IG, Fisher G, Flohr P, Reid A, Edwards S, Kovacs G, Berney D, Foster C, Massie CE, Fletcher A, De Bono JS, Scardino P, Cuzick J, Cooper CS (2008c) Heterogeneity and clinical significance of ETV1 translocations in human prostate cancer. Br J Cancer 99: 314–320 18594527 10.1038/sj.bjc.6604472 PMC2480965

[bib4] Bedolla R, Prihoda TJ, Kreisberg JI, Malik SN, Krishnegowda NK, Troyer DA, Ghosh PM (2007) Determining risk of biochemical recurrence in prostate cancer by immunohistochemical detection of PTEN expression and Akt activation. Clin Cancer Res 13: 3860–3867 17606718 10.1158/1078-0432.CCR-07-0091

[bib5] Betensky RA, Louis DN, Cairncross JG (2002) Influence of unrecognized molecular heterogeneity on randomized clinical trials. J Clin Oncol 20: 2495–2499 12011127 10.1200/JCO.2002.06.140

[bib6] Carver BS, Tran J, Gopalan A, Chen Z, Shaikh S, Carracedo A, Alimonti A, Nardella C, Varmeh S, Scardino PT, Cordon-Cardo C, Gerald W, Pandolfi PP (2009) Aberrant ERG expression cooperates with loss of PTEN to promote cancer progression in the prostate. Nat Genet 41: 619–624 19396168 10.1038/ng.370 PMC2835150

[bib7] Clark JP, Cooper CS (2009) ETS gene fusions in prostate cancer. Nat Rev Urol 6: 429–439 19657377 10.1038/nrurol.2009.127

[bib8] Cox D, Oakes D (1984) Analysis of Survival Data. Chapman and Hall: London, New York

[bib9] Cuzick J, Fisher G, Kattan MW, Berney D, Oliver T, Foster CS, Moller H, Reuter V, Fearn P, Eastham J, Scardino P (2006) Long-term outcome among men with conservatively treated localised prostate cancer. Br J Cancer 95: 1186–1194 17077805 10.1038/sj.bjc.6603411 PMC2360576

[bib10] Demichelis F, Fall K, Perner S, Andren O, Schmidt F, Setlur SR, Hoshida Y, Mosquera JM, Pawitan Y, Lee C, Adami HO, Mucci LA, Kantoff PW, Andersson SO, Chinnaiyan AM, Johansson JE, Rubin MA (2007) TMPRSS2:ERG gene fusion associated with lethal prostate cancer in a watchful waiting cohort. Oncogene 26: 4596–4599 17237811 10.1038/sj.onc.1210237

[bib11] Ferlay J, Autier P, Boniol M, Heanue M, Colombet M, Boyle P (2007) Estimates of the cancer incidence and mortality in Europe in 2006. Ann Oncol 18: 581–592 17287242 10.1093/annonc/mdl498

[bib12] FitzGerald LM, Agalliu I, Johnson K, Miller MA, Kwon EM, Hurtado-Coll A, Fazli L, Rajput AB, Gleave ME, Cox ME, Ostrander EA, Stanford JL, Huntsman DG (2008) Association of TMPRSS2-ERG gene fusion with clinical characteristics and outcomes: results from a population-based study of prostate cancer. BMC Cancer 8: 230 18694509 10.1186/1471-2407-8-230 PMC2519091

[bib13] Gopalan A, Leversha MA, Satagopan JM, Zhou Q, Al-Ahmadie HA, Fine SW, Eastham JA, Scardino PT, Scher HI, Tickoo SK, Reuter VE, Gerald WL (2009) TMPRSS2-ERG gene fusion is not associated with outcome in patients treated by prostatectomy. Cancer Res 69: 1400–1406 19190343 10.1158/0008-5472.CAN-08-2467 PMC3676271

[bib14] Gouyer V, Fontaine D, Dumont P, de Wever O, Fontayne-Devaud H, Leteurtre E, Truant S, Delacour D, Drobecq H, Kerckaert JP, de Launoit Y, Bracke M, Gespach C, Desseyn JL, Huet G (2008) Autocrine induction of invasion and metastasis by tumor-associated trypsin inhibitor in human colon cancer cells. Oncogene 27: 4024–4033 18317448 10.1038/onc.2008.42

[bib15] Hermans KG, Boormans JL, Gasi D, van Leenders GJ, Jenster G, Verhagen PC, Trapman J (2009) Overexpression of prostate-specific TMPRSS2(exon 0)-ERG fusion transcripts corresponds with favorable prognosis of prostate cancer. Clin Cancer Res 15: 6398–6403 19825963 10.1158/1078-0432.CCR-09-1176

[bib16] Hermans KG, van Alewijk DC, Veltman JA, van Weerden W, van Kessel AG, Trapman J (2004) Loss of a small region around the PTEN locus is a major chromosome 10 alteration in prostate cancer xenografts and cell lines. Genes Chromosomes Cancer 39: 171–184 14732919 10.1002/gcc.10311

[bib17] King JC, Xu J, Wongvipat J, Hieronymus H, Carver BS, Leung DH, Taylor BS, Sander C, Cardiff RD, Couto SS, Gerald WL, Sawyers CL (2009) Cooperativity of TMPRSS2-ERG with PI3-kinase pathway activation in prostate oncogenesis. Nat Genet 41: 524–526 19396167 10.1038/ng.371 PMC2898503

[bib18] Kirkegaard T, Edwards J, Tovey S, McGlynn LM, Krishna SN, Mukherjee R, Tam L, Munro AF, Dunne B, Bartlett JM (2006) Observer variation in immunohistochemical analysis of protein expression, time for a change? Histopathology 48: 787–794 16722926 10.1111/j.1365-2559.2006.02412.x

[bib19] Kumar-Sinha C, Tomlins SA, Chinnaiyan AM (2008) Recurrent gene fusions in prostate cancer. Nat Rev Cancer 8: 497–511 18563191 10.1038/nrc2402 PMC2711688

[bib20] McCall P, Witton CJ, Grimsley S, Nielsen KV, Edwards J (2008) Is PTEN loss associated with clinical outcome measures in human prostate cancer? Br J Cancer 99: 1296–1301 18854827 10.1038/sj.bjc.6604680 PMC2570524

[bib21] Nam RK, Sugar L, Yang W, Srivastava S, Klotz LH, Yang LY, Stanimirovic A, Encioiu E, Neill M, Loblaw DA, Trachtenberg J, Narod SA, Seth A (2007) Expression of the TMPRSS2:ERG fusion gene predicts cancer recurrence after surgery for localised prostate cancer. Br J Cancer 97: 1690–1695 17971772 10.1038/sj.bjc.6604054 PMC2360284

[bib22] Petrovics G, Liu A, Shaheduzzaman S, Furusato B, Sun C, Chen Y, Nau M, Ravindranath L, Chen Y, Dobi A, Srikantan V, Sesterhenn IA, McLeod DG, Vahey M, Moul JW, Srivastava S (2005) Frequent overexpression of ETS-related gene-1 (ERG1) in prostate cancer transcriptome. Oncogene 24: 3847–3852 15750627 10.1038/sj.onc.1208518

[bib23] Sircar K, Yoshimoto M, Monzon FA, Koumakpayi IH, Katz RL, Khanna A, Alvarez K, Chen G, Darnel AD, Aprikian AG, Saad F, Bismar TA, Squire JA (2009) PTEN genomic deletion is associated with p-Akt and AR signalling in poorer outcome, hormone refractory prostate cancer. J Pathol 218(4): 505–513 19402094 10.1002/path.2559

[bib24] Tomlins SA, Laxman B, Dhanasekaran SM, Helgeson BE, Cao X, Morris DS, Menon A, Jing X, Cao Q, Han B, Yu J, Wang L, Montie JE, Rubin MA, Pienta KJ, Roulston D, Shah RB, Varambally S, Mehra R, Chinnaiyan AM (2007) Distinct classes of chromosomal rearrangements create oncogenic ETS gene fusions in prostate cancer. Nature 448: 595–599 17671502 10.1038/nature06024

[bib25] Tomlins SA, Laxman B, Varambally S, Cao X, Yu J, Helgeson BE, Cao Q, Prensner JR, Rubin MA, Shah RB, Mehra R, Chinnaiyan AM (2008a) Role of the TMPRSS2-ERG gene fusion in prostate cancer. Neoplasia 10: 177–188 18283340 10.1593/neo.07822 PMC2244693

[bib26] Tomlins SA, Rhodes DR, Perner S, Dhanasekaran SM, Mehra R, Sun XW, Varambally S, Cao X, Tchinda J, Kuefer R, Lee C, Montie JE, Shah RB, Pienta KJ, Rubin MA, Chinnaiyan AM (2005) Recurrent fusion of TMPRSS2 and ETS transcription factor genes in prostate cancer. Science 310: 644–648 16254181 10.1126/science.1117679

[bib27] Tomlins SA, Rhodes DR, Yu J, Varambally S, Mehra R, Perner S, Demichelis F, Helgeson BE, Laxman B, Morris DS, Cao Q, Cao X, Andren O, Fall K, Johnson L, Wei JT, Shah RB, Al-Ahmadie H, Eastham JA, Eggener SE, Fine SW, Hotakainen K, Stenman UH, Tsodikov A, Gerald WL, Lilja H, Reuter VE, Kantoff PW, Scardino PT, Rubin MA, Bjartell AS, Chinnaiyan AM (2008b) The role of SPINK1 in ETS rearrangement-negative prostate cancers. Cancer Cell 13: 519–528 18538735 10.1016/j.ccr.2008.04.016 PMC2732022

[bib28] Trotman LC, Niki M, Dotan ZA, Koutcher JA, Di Cristofano A, Xiao A, Khoo AS, Roy-Burman P, Greenberg NM, Van Dyke T, Cordon-Cardo C, Pandolfi PP (2003) Pten dose dictates cancer progression in the prostate. PLoS Biol 1: E59 14691534 10.1371/journal.pbio.0000059 PMC270016

[bib29] Ventura RA, Martin-Subero JI, Jones M, McParland J, Gesk S, Mason DY, Siebert R (2006) FISH analysis for the detection of lymphoma-associated chromosomal abnormalities in routine paraffin-embedded tissue. J Mol Diagn 8: 141–151 16645199 10.2353/jmoldx.2006.050083 PMC1867591

[bib30] Verhagen PC, van Duijn PW, Hermans KG, Looijenga LH, van Gurp RJ, Stoop H, van der Kwast TH, Trapman J (2006) The PTEN gene in locally progressive prostate cancer is preferentially inactivated by bi-allelic gene deletion. J Pathol 208: 699–707 16402365 10.1002/path.1929

[bib31] Whang YE, Wu X, Suzuki H, Reiter RE, Tran C, Vessella RL, Said JW, Isaacs WB, Sawyers CL (1998) Inactivation of the tumor suppressor PTEN/MMAC1 in advanced human prostate cancer through loss of expression. Proc Natl Acad Sci USA 95: 5246–5250 9560261 10.1073/pnas.95.9.5246 PMC20246

[bib32] Yap TA, Garrett MD, Walton MI, Raynaud F, de Bono JS, Workman P (2008) Targeting the PI3K-AKT-mTOR pathway: progress, pitfalls, and promises. Curr Opin Pharmacol 8: 393–412 18721898 10.1016/j.coph.2008.08.004

[bib33] Yoshimoto M, Cunha IW, Coudry RA, Fonseca FP, Torres CH, Soares FA, Squire JA (2007) FISH analysis of 107 prostate cancers shows that PTEN genomic deletion is associated with poor clinical outcome. Br J Cancer 97(5): 678–685 17700571 10.1038/sj.bjc.6603924 PMC2360375

[bib34] Yoshimoto M, Joshua AM, Cunha IW, Coudry RA, Fonseca FP, Ludkovski O, Zielenska M, Soares FA, Squire JA (2008) Absence of TMPRSS2:ERG fusions and PTEN losses in prostate cancer is associated with a favorable outcome. Mod Pathol 21: 1451–1460 18500259 10.1038/modpathol.2008.96

