# Supplementary figures and images for: Molecular characterisation of ERG, ETV1 and PTEN gene loci identifies patients at low and high risk of death from prostate cancer
Source: Br J Cancer. 2010 Jan 26;102(4):678–84. doi: 10.1038/sj.bjc.6605554 (PMC2837564; doi:10.1038/sj.bjc.6605554)

## Slide 1
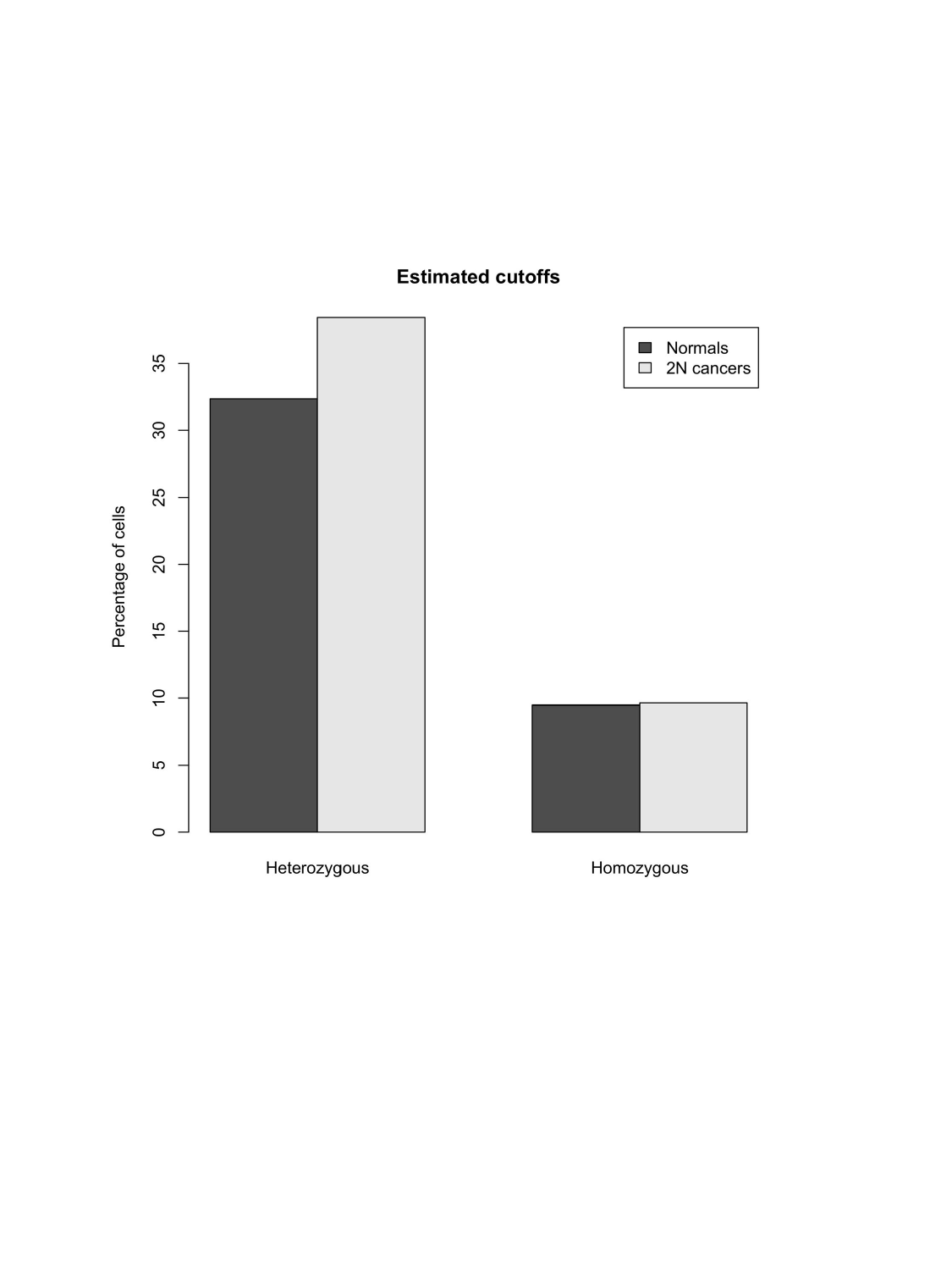

Supplement: Supplementary Figure 1 [file 6605554x1.ppt]
