# Supplementary material for: Molecular characterisation of ERG, ETV1 and PTEN gene loci identifies patients at low and high risk of death from prostate cancer
Source: Br J Cancer. 2010 Jan 26;102(4):678–84. doi: 10.1038/sj.bjc.6605554 (PMC2837564; doi:10.1038/sj.bjc.6605554)
Supplement: Supplementary Figure Legend [file 6605554x2.doc]

**Supplementary Figure 1: Calculating heterozygous and homozygous cut-offs.** From the data in Supplementary Table 1, the mean number of nuclei with a heterozygous and homozygous *PTEN* loss pattern was calculated and 3 standard deviations (SD) added. The bar chart demonstrates that the mean percentage ± 3 S.D of normal prostate epithelial cells (dark grey) with apparent heterozygous and homozygous loss was 32.4% (left- dark grey) and 9.4% (right- dark grey) respectively. The mean percentage ± 3 S.D for cancer epithelial cells with a ‘Normal’ PTEN complement (light grey) with apparent heterozygous and homozygous loss was 38.4% (left-light grey) and 9.5% (right-light grey) respectively (p < 0.0015). Therefore to classify a TMA cancer core as having homozygous *PTEN* loss, simultaneous lack of both signals of *PTEN* gene locus-targeted probe and the presence of 2 signals of chromosome 10 centromeric probe had to occur in ≥ 10% of nuclei. To classify a core as having heterozygous loss ≥ 40% of nuclei had to contain one signal of *PTEN* gene locus-targeted probe and 2 signals of chromosome 10 centromeric probes. Occasionally ploidy was seen and deletions were relative rather than absolute. For example, 2 signals of locus-targeted probe and 4 signals of reference probe were scored as a heterozygous loss.
