# Supplementary material for: Molecular characterisation of ERG, ETV1 and PTEN gene loci identifies patients at low and high risk of death from prostate cancer
Source: Br J Cancer. 2010 Jan 26;102(4):678–84. doi: 10.1038/sj.bjc.6605554 (PMC2837564; doi:10.1038/sj.bjc.6605554)
Supplement: Supplementary Table 1 [file 6605554x3.doc]

| **Tumour core** | **2 green/ 1 red**  **probes** | **2 red/ 1 green**  **(Hetero)** | **2 red probes (Homo)** | **2 green probes** | **2 red + 2 green**  **(Normal)** | **1 red + 1 green**  **probe** | **1 red probe** | **1 green probe** |
| --- | --- | --- | --- | --- | --- | --- | --- | --- |
| **1** | 6 | 11 | 1 | 2 | 28 | 38 | 6 | 8 |
| **2** | 8 | 12 | 6 | 1 | 31 | 33 | 5 | 4 |
| **3** | 6 | 12 | 1 | 1 | 30 | 39 | 9 | 2 |
| **4** | 11 | 9 | 0 | 3 | 40 | 29 | 5 | 3 |
| **5** | 9 | 12 | 2 | 1 | 37 | 30 | 1 | 8 |
| **6** | 25 | 24 | 1 | 2 | 85 | 47 | 9 | 7 |
| **7** | 20 | 25 | 7 | 1 | 60 | 72 | 9 | 6 |
| **8** | 21 | 10 | 2 | 2 | 86 | 74 | 2 | 3 |
| **9** | 23 | 18 | 2 | 3 | 56 | 63 | 18 | 17 |
| **10** | 23 | 24 | 11 | 4 | 36 | 66 | 21 | 15 |
