# Supplementary material for: Molecular characterisation of ERG, ETV1 and PTEN gene loci identifies patients at low and high risk of death from prostate cancer
Source: Br J Cancer. 2010 Jan 26;102(4):678–84. doi: 10.1038/sj.bjc.6605554 (PMC2837564; doi:10.1038/sj.bjc.6605554)
Supplement: Supplementary Table Legend [file 6605554x4.doc]

**Supplementary Table 1: Counting FISH signals.** PTEN (green) and chromosome 10 centromere (red) probe patterns were counted in 5 TMA cores of normal prostate epithelial cells (rows 1-5) and in 5 cores of prostate cancer epithelial cells (rows 6-10). The probe patterns are described at the top of each column and the number of nuclei with that pattern listed beneath for each of the TMA cores. Values in rows 1-5 (in black) show probe pattern counts in normal prostate epithelial cells (100 cells counted in each of the 5 cores). Values in rows 6-10 (in red) show probe pattern counts in prostate cancer epithelial cells designated as having a ‘Normal’ PTEN complement (200 cells counted in each of the 5 cores). There were no cores with ploidy in this analysis. ‘Hetero’ refers to heterozygous and ‘homo’ to homozygous loss of *PTEN*.
